# Impacts of Climate Change and Inter-Specific Competition on the Spatial Distribution of Elliot’s Pheasant (*Syrmaticus ellioti*, Swinhoe, 1872) in Huzhou City, China

**DOI:** 10.3390/biology15060480

**Published:** 2026-03-18

**Authors:** Yongxiang Zhao, Xiaofan Jiang, Min Jiang, Yongqiang Qin, Yue Song, Yujie Zhang, Ke He, Liqiong Peng

**Affiliations:** 1 Huzhou Ecological Forestry Protection Research Center, Huzhou 313000, China; 2 Changxing County Forestry Technology Extension Center, Huzhou 313100, China; 3 College of Animal Science and Technology & College of Veterinary Medicine, Zhejiang Agriculture and Forestry University, Hangzhou 311300, China

**Keywords:** Elliot’s pheasant (*Syrmaticus ellioti*), ground-dwelling pheasants, species distribution model, MaxEnt, niche overlap, climate change, infrared camera monitoring

## Abstract

Ground-dwelling pheasants are vital indicators of forest ecosystem health. Understanding their distribution and response to climate change is crucial for regional biodiversity conservation. Based on 97,000 camera-days of infrared monitoring from 2019 to 2022 in Huzhou, China, we analyzed the spatial patterns and niche overlap of five pheasant species, including the first class national protected animal Elliot’s Pheasant (*Syrmaticus ellioti*), using MaxEnt modeling and Schoener’s D index. Results showed the following: (1) Pheasants in Huzhou exhibited distinct vertical gradients, with Elliot’s Pheasant restricted primarily to mid-mountain forests (200–600 m) in western Anji. (2) Iso-thermality and winter thermal limits were the primary drivers of its distribution. (3) Niche analysis revealed intense competitive pressure; Elliot’s Pheasant habitat was largely encompassed by dominant species like the Silver Pheasant (*Lophura nycthemera*), showing a high overlap (D = 0.642) with the Koklass Pheasant (*Pucrasia macrolopha*). (4) By 2050, its suitable habitat is projected to shrink by 84.6% (from 1085.7 to 118.8 km^2^) and shift eastward. These findings highlight the high climate sensitivity and competitive vulnerability of Elliot’s Pheasant. We recommend prioritizing micro-habitat maintenance in mid-mountain zones and proactively establishing ecological corridors between Anji and Deqing to mitigate habitat loss and displacement.

## Simple Summary

Ground-dwelling pheasants are essential indicators of forest health, yet they face increasing threats from environmental instability. This study utilized city-wide infrared camera monitoring to assess the spatial ecology and competitive dynamics of the endangered Elliot’s Pheasant in Huzhou, China. Our analysis identifies the species as a climate-sensitive specialist, restricted by specific thermal requirements and facing intense competitive exclusion from more resilient sympatric neighbors. Projections for 2050 indicate a catastrophic 84.6% habitat loss and significant range shifts due to warming. These findings highlight a critical population bottleneck. To ensure long-term persistence, we advocate for the preservation of mid-elevation micro-habitats and the strategic implementation of ecological corridors to facilitate climate-driven migration and maintain regional biodiversity.

## 1. Introduction

Global climate change has emerged as one of the most critical challenges to biodiversity conservation in the 21st century. The resulting environmental fluctuations are profoundly altering global species distribution patterns, leaving approximately one million animal and plant species at risk of extinction [1]. Within the context of climate change, unsustainable human activities—including habitat destruction, fragmentation, and overexploitation—have further constrained the living space of endemic species [2]. Consequently, accurately predicting and understanding how species respond to environmental shifts has become a prerequisite for developing effective conservation interventions [3]. Species distribution models provide essential technical support for identifying suitable habitats and predicting distributional shifts by quantifying the non-linear relationships between biological occurrence points and environmental factors. Among these, the Maximum Entropy (MaxEnt) model is widely utilized in conservation planning for rare species due to its robustness and high predictive accuracy when processing limited sample sizes [4,5].

The Elliot’s Pheasant (*Syrmaticus ellioti*) is a typical ground-dwelling forest bird endemic to China. It is currently listed as Near Threatened (NT) on the IUCN Red List and is recognized as a first-class national protected animal in China. This species primarily inhabits various environments between elevations of 200 and 1900 m in Zhejiang, Fujian, Jiangxi, Anhui, Hunan, Guizhou, Guangxi, and Guangdong Provinces, such as evergreen broad-leaved forests and coniferous–broadleaf mixed forests, with broad-leaved and mixed forests being particularly vital [6]. Research indicates that Elliot’s Pheasant exhibits high selectivity in habitat utilization; canopy cover, concealment conditions, and distance to water sources are core factors determining its distribution [7,8,9,10]. Furthermore, its distribution displays significant seasonal dynamics: in the Gutianshan National Nature Reserve, the population tends to migrate toward higher altitudes as mean monthly temperatures rise, with activity intensity and range in summer and autumn being notably greater than in winter and spring [9]. The species faces extinction risks in localized areas due to multiple stressors, including high habitat fragmentation [11], shrinking suitable habitat area, and genetic bottleneck effects [12].

At the community level, Elliot’s Pheasant often occurs sympatrically with species such as the Silver Pheasant (*Lophura nycthemera*) and the Koklass Pheasant (*Pucrasia macrolopha*). According to niche theory, sympatric species must reduce inter-specific resource competition through differentiation in at least one dimension—habitat, food, or time—to achieve stable coexistence [13]. Given that pheasants generally exhibit low mobility and limited spatial movement capabilities, their niches often overlap significantly, leading to intense inter-specific competition. In the Fanjingshan National Nature Reserve, elevation is the primary factor influencing pheasant occupancy, with the Temminck’s Tragopan (*Tragopan temminckii*) showing different elevational preferences compared to three other pheasant species [14]. Similarly, in the Jiangxi Guanshan National Nature Reserve, four sympatric pheasant species also exhibit differences in their relatively concentrated elevational distributions [15]. Regarding temporal niches, research in the Qingliangfeng National Nature Reserve found that although these pheasants are diurnal animals primarily active during dawn and dusk—resulting in high overlap coefficients for daily activity rhythms throughout the year—their peak activity periods do not completely coincide [16]. Under such high competitive pressure, the maintenance of population stability and community status for Elliot’s Pheasant requires support from diverse ecological strategies. Despite its protected status, most existing research and conservation efforts are concentrated within established nature reserves, leaving a critical knowledge gap regarding population dynamics and habitat connectivity in human-dominated or non-protected landscapes. This study addresses this conservation blind spot by focusing on a significant but under-surveyed region in Huzhou City, Zhejiang Province, providing essential data to evaluate the importance of non-protected habitats for the species’ long-term survival. While Huzhou has established a baseline for rare species through grid-based infrared camera surveys, there remains a lack of continuous monitoring data regarding how cryptic threatened species respond to climate fluctuations and inter-specific competition at local scales. Therefore, this research aims to (1) identify the core environmental factors driving the spatial distribution of Elliot’s Pheasant in the region; (2) assess niche overlap with sympatric pheasant species to elucidate survival pressures and habitat constraints under strong competition; and (3) simulate changes in suitable habitat area and spatial shifts of core areas under different climate change scenarios. This study aimed to provide scientific evidence to support the proactive layout of trans-regional protection and the formulation of precision conservation strategies for Elliot’s Pheasant in response to climate change.

## 2. Materials and Methods

### 2.1. Data Sources

**Figure 1:**
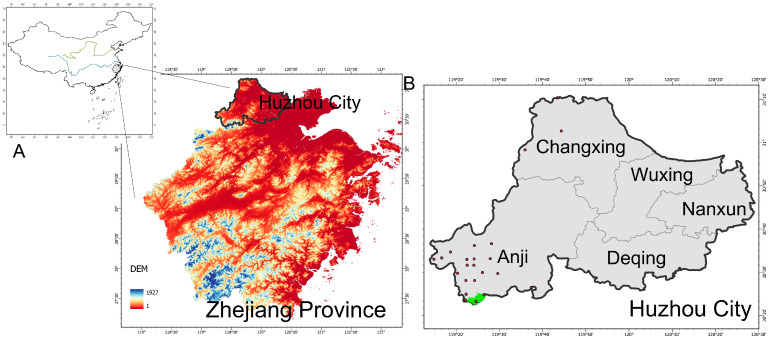
Geographical location of the study area. (**A**) The regional extent of Zhejiang Province used for model construction; (**B**) map of Huzhou City showing the infrared camera observation points of *Syrmaticus ellioti* (red dots), with the green shaded area representing the Anji Salamander National Nature Reserve.

The study area, Huzhou City, is situated in northern Zhejiang Province (Figure 1). The terrain transitions from high elevations in the west to lower elevations in the east, dominated by plains and hills under a north subtropical monsoon climate. To address the lack of continuous monitoring for cryptic threatened species at local scales, we utilized long-term monitoring data obtained via a comprehensive city-wide infrared camera network. From 2019 to 2022, infrared camera technology was employed to survey mammals and ground-dwelling birds across Changxing County, Anji County, Deqing County, and Wuxing District. Cameras were deployed using grid-based sampling with parameters adjusted according to local habitat characteristics and geographic conditions. Additionally, occurrence data for the target species were supplemented from the Global Biodiversity Information Facility (GBIF, http://www.gbif.org/, https://doi.org/10.15468/dl.ujz4wx for Elliot’s Pheasant, https://doi.org/10.15468/dl.cpqt5e for Silver Pheasant, https://doi.org/10.15468/dl.x9hqj3 for Koklass Pheasant, https://doi.org/10.15468/dl.kszwpu for Chinese Bamboo Partridge (*Bambusicola thoracicus*), accessed on 2 February 2026) and another survey in Zhejiang Province [17]. Five ground-dwelling pheasant species were included: Elliot’s Pheasant, Silver Pheasant, Koklass Pheasant, Chinese Bamboo Partridge, and Common Pheasant (*Phasianus colchicus*).

To mitigate spatial correlation and ensure consistency with the environmental factor resolution, occurrence points from the Huzhou monitoring work and GBIF were overlaid and filtered using ENMTools (v1.3). We applied a spatial filtering threshold of 30″ to remove redundant data points, ensuring that the filtered occurrence records match the spatial resolution of the environmental variables. Redundant data points were removed to produce the final dataset for subsequent analysis.

### 2.2. Environmental Variables

Four categories of ecological factors—climate, topography, vegetation, and human disturbance—were selected as candidate environmental variables for predicting species distribution. All raster data were resampled to a spatial resolution of 30″ (approximately 1 km).

(1) Climatic factors: Nineteen bioclimatic variables (Bio01–Bio19) for the current period and the future period (2040–2060) were obtained from the WorldClim database (http://www.worldclim.org/ (accessed on 2 February 2026)). Future projections utilized the latest Shared Socioeconomic Pathway (SSP2-4.5) from the IPCC AR6. These factors reflect extremes, seasonality, and inter-annual variations in temperature and precipitation, serving as core determinants of macro-scale distributions.
(2) Vegetation and topographic factors: The Normalized Difference Vegetation Index (NDVI, from https://modis.gsfc.nasa.gov/ (accessed on 2 February 2026)) was utilized as a key indicator of vegetation cover and habitat quality, representing food availability and concealment conditions. We calculated the annual mean NDVI from monthly datasets to represent long-term habitat quality. Three topographic factors—elevation (Alt), slope (Slope), and aspect (Aspect)—were extracted from a Digital Elevation Model (DEM). These factors influence micro-habitat selection by regulating the redistribution of local heat and moisture.
(3) Human disturbance: To quantitatively assess the impact of anthropogenic activities, the Global Human Footprint Index (HFP) was sourced from the Socioeconomic Data and Applications Center (htps://sedac.ciesin.columbia.edu/data/ (accessed on 2 February 2026)) at Columbia University. The HFP systematically characterizes the spatial patterns of human intensity and the resulting pressure on natural habitats.

### 2.3. Selection of Environmental Variables

**Figure 2:**
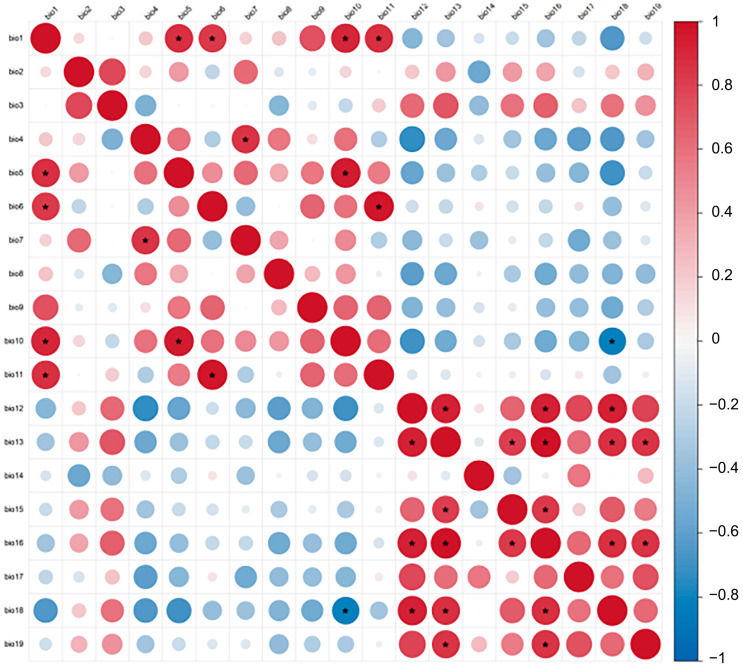
Correlation analysis of climate factors in the studied area. * indicates |r| > 0.8.

High multi-collinearity among climatic factors can lead to model over-fitting; therefore, a screening process was conducted. A Pearson correlation test was performed on the 19 bioclimatic variables. For pairs exhibiting strong correlation (|r| > 0.80), the variable with the lower contribution to the preliminary model was excluded (Figure 2 and Figure S1). Topographic factors were assumed to remain constant throughout the future projection periods.

### 2.4. Model Construction and Suitability Classification

The MaxEnt model was employed to simulate the potential geographic distribution of the species. To overcome the statistical limitations posed by the limited sample size within the local study area, occurrence records across the entire Zhejiang Province were used to train the model, ensuring a precise characterization of the species’ ecological niche. Subsequently, the calibrated model was projected onto Huzhou City to simulate its local distribution. For the modeling process, the screened geographic, climatic, and anthropogenic factors were imported as explanatory variables. The dataset was partitioned, with 75% of occurrence points used for model training and the remaining 25% reserved for performance evaluation. To eliminate bias from random sampling, the model was executed for 10 replicates using the Bootstrap method, and the average results were recorded; all other parameters were kept at system defaults. The Jack-knife test was used to evaluate the contribution and importance of each environmental variable, identifying the dominant ecological factors limiting spatial distribution.

To minimize multicollinearity and ensure model stability, we selected variables based on their ecological relevance and initial contribution (Table 1) and conducted a Variance Inflation Factor (VIF) analysis on these candidates, using the “usdm” package in R.

Using ArcGIS Pro (3.0.2) and the Natural Breaks (Jenks) classification method, habitat suitability was categorized into four levels: unsuitable, low suitability, moderate suitability, and high suitability. This method was selected for its ability to identify natural groupings in the data by minimizing the sum of squared deviations within each class, thereby ensuring that the habitat thresholds are statistically objective.

### 2.5. Niche Overlap Analysis of Sympatric Pheasants

To assess the inter-specific competition and resource partitioning, we quantified the ecological niche overlap between Elliot’s Pheasant and its sympatric pheasant species using the Schoener’s D index in the “ecospat” R package (version 4). This analysis was based on habitat suitability models, which account for underlying environmental gradients. To determine whether the observed niche overlap differed significantly from random expectations, we conducted niche equivalency and similarity tests with 100 random permutations. Second, spatial intersection areas were extracted in ArcGIS based on the suitability results predicted by the MaxEnt models. By analyzing the spatial overlap, the study further explored the spatial utilization strategies and niche differentiation characteristics of these taxonomically or ecologically similar species within limited habitats.

## 3. Results

### 3.1. Distribution Overview of Pheasants in Huzhou

A systematic infrared camera monitoring program was conducted across the entirety of Huzhou. A total of 485 infrared cameras were deployed, primarily targeting forest habitats while also covering various other vegetation types. During the survey period, the cumulative effective camera working days exceeded 97,000, yielding approximately 388,000 raw images and over 61,000 independent effective records.

The monitoring results revealed significant differences in the distribution patterns of the five pheasant species. The Chinese Bamboo Partridge recorded the highest frequency, appearing at 153 monitoring sites with 1740 independent photographs. The Silver Pheasant exhibited the widest spatial coverage, recorded at 112 sites with 704 independent photographs. Among the other species, the Koklass Pheasant occurred at 71 sites (333 photographs), and Elliot’s Pheasant was recorded at 23 sites (248 photographs). In contrast, the Common Pheasant showed the most restricted distribution, recorded at only 20 sites. Due to this limited number of occurrences, the Common Pheasant was excluded from subsequent modeling analysis.

### 3.2. Results of Environmental Variable Selection

**Figure 3:**
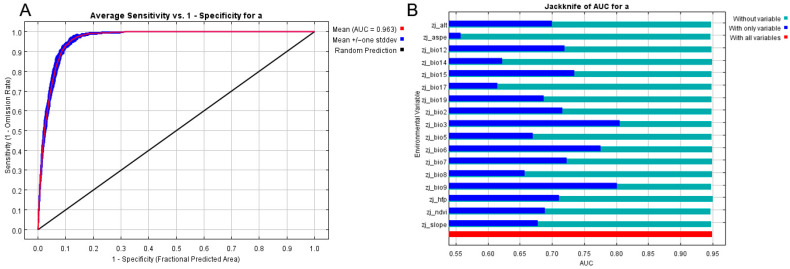
(**A**) Maxent model analysis results and (**B**) the influence of related ecological factors for *Syrmaticus ellioti*.

Prior to model construction, a correlation analysis was performed on the 19 initial bioclimatic factors (Figure 2). Based on the Pearson correlation coefficient (|r| < 0.8) and the biological relevance of each variable to pheasant distribution, 11 core climatic factors were selected for the MaxEnt analysis (Results in Table 1 and Figure 3). These factors represent key extremes and seasonal variations in temperature and precipitation: mean diurnal range (Bio02), max temperature of warmest month (Bio05), min temperature of coldest month (Bio06), temperature annual range (Bio07), mean temperature of wettest quarter (Bio08), mean temperature of driest quarter (Bio09), annual precipitation (Bio12), precipitation of driest month (Bio14), precipitation seasonality (Bio15), precipitation of driest quarter (Bio17), and precipitation of coldest quarter (Bio19).

### 3.3. Model Accuracy and Contribution of Environmental Factors

#### 3.3.1. Predictive Accuracy of the Model

The potential distribution patterns of the four pheasant species in Huzhou were simulated using MaxEnt. The model accuracy results are presented in Table 2. The Area Under the Curve (AUC) values for the training sets were high: 0.963 for Elliot’s Pheasant, 0.961 for the Koklass Pheasant, and 0.909 for the Silver Pheasant, all indicating excellent predictive performance. The AUC for the Chinese Bamboo Partridge was 0.859, reflecting good performance. These results suggest that the selected environmental variables effectively explained the spatial distribution characteristics of these species. Iterative screening of 16 initial ecological factors revealed that thermal stability, elevation, and seasonal moisture conditions were the core drivers of pheasant distribution, though species exhibited both differentiation and convergence in their response characteristics.

#### 3.3.2. Impact of Environmental Factors on Elliot’s Pheasant

The distribution of Elliot’s Pheasant was modeled using a subset of variables that achieved statistical independence (VIF < 2). The model was primarily driven by iso-thermality (Bio03, 26.6%), elevation (Alt, 18.0%), and precipitation seasonality (Bio15, 14.7%), which collectively contributed nearly 60%. Response curve analysis indicated a significant preference for stable micro-climates, with the suitable range for Bio03 concentrated between 22 and 26 °C. Additionally, the permutation importance of mean temperature of driest quarter (Bio09) reached 31.7%—the highest in the model. Given that our VIF analysis confirms that this result is not an artifact of multicollinearity, Bio09 appears to be a critical limiting factor for the species, suggesting a preference for leeward forest edges with optimal winter thermal conditions (2–6 °C). Spatially, elevation defined its vertical distribution center within the 200–600 m low-to-middle mountain range, with a preference for sloped terrain and complex vertical vegetation structures for concealment. The HFP showed a significant negative correlation with occurrence probability, confirming high sensitivity to habitat integrity. Its core habitat is characterized by low-to-middle elevations, stable temperature ranges, and minimal human interference.

#### 3.3.3. Impact of Environmental Factors on the Other Three Pheasant Species

Model results for the other three sympatric pheasants also showed strong constraints by thermal stability and topography. Silver and Koklass Pheasants relied heavily on environmental stability, with Bio03 contributions of 33.0% and 21.8%, respectively. The Chinese Bamboo Partridge was more limited by Temperature Annual Range (Bio07, 27.1%), with suitability decreasing as the temperature range increased. All three species exhibited distinct threshold effects regarding winter heat, with Bio09 serving as the critical factor for over-wintering habitat selection. Vertically, elevation played a regulatory role: the Koklass Pheasant’s core range was situated in the 400–1000 m mid-mountain zone, showing strong high-altitude adaptation. Conversely, the Silver Pheasant and Chinese Bamboo Partridge were concentrated in the 200–600 m low-mountain and hilly zones. While these species overlapped in thermal niches, spatial differentiation was achieved through altitudinal gradients. All species responded negatively to increased human footprints, highlighting the biological importance of protecting contiguous natural and secondary forests.

### 3.4. Analysis of Niche Overlap and Spatial Competition

#### 3.4.1. Habitat Constraints and Spatial Characteristics of Elliot’s Pheasant

**Figure 4:**
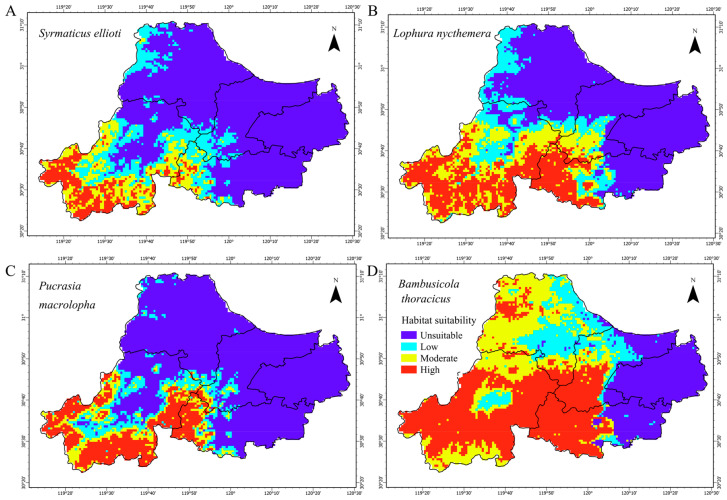
Distribution of suitable habitats for (**A**) *Syrmaticus ellioti*, (**B**) *Lophura nycthemera*, (**C**) *Pucrasia macrolopha*, and (**D**) *Bambusicola thoracicus* under current climate conditions.

As the focal species of this assessment, Elliot’s Pheasant exhibited extreme habitat specificity and restricted distribution in Huzhou (Figure 4). Simulations showed that its suitable habitat (moderate and high suitability) totaled 1086.9 km^2^, accounting for only 18.7% of the study area (Table 2). Unsuitable habitat covered 3820.0 km^2^, the second-largest among the four species after the Koklass Pheasant. Infrared monitoring further confirmed this fragmentation; the species was recorded at only 23 sites, with a grid occupancy rate of 7.3%. Distribution was highly restricted to specific areas in Anji and Changxing Counties, while the species was absent in Deqing and Wuxing. The high-suitability area (434.8 km^2^) was concentrated in the western mountains of Anji. Kernel density analysis identified the Anji Salamander National Nature Reserve as the core population stronghold, with local densities reaching 1.42–1.68 individuals/km^2^. This tight spatial layout reflects a strict reliance on low-interference forest interiors.

#### 3.4.2. Altitudinal Gradients and Thermal Niche Differentiation

A comparison of the four species revealed a significant tiered distribution along environmental gradients. Vertically, the Koklass Pheasant occupied high-altitude zones (400–1000 m) and remained viable above 800 m. In contrast, the Chinese Bamboo Partridge (100–450 m) and Silver Pheasant (150–500 m) preferred lower hills. Elliot’s Pheasant occupied the middle tier (200–600 m). Regarding thermal tolerance, the Koklass Pheasant was the most cold-tolerant (Bio09 threshold down to 1 °C), while the Silver Pheasant and Chinese Bamboo Partridge required warmer winter temperatures. Notably, Elliot’s Pheasant and the Silver Pheasant both showed high dependency on Bio03, indicating a shared reliance on closed-canopy secondary forests with strong microclimate regulation.

#### 3.4.3. Spatial Overlap and Competition Based on the D-Index

The niche overlap (Schoener’s D) between Elliot’s Pheasant and its counterparts was 0.684 (vs. Silver Pheasant), 0.642 (vs. Koklass Pheasant), and 0.407 (vs. Chinese Bamboo Partridge). Statistical significance tests revealed that the niche overlap between Elliot’s Pheasant and the Silver Pheasant was significantly higher than random expectations (similarity test, *p* < 0.01), suggesting a pattern of niche convergence. In contrast, the equivalency tests for all species pairs yielded non-significant results (*p* > 0.05), indicating that the niches occupied by Elliot’s Pheasant are statistically equivalent to those of its sympatric species. These findings underscore that Elliot’s Pheasant faces the most intense competitive pressure from the Silver Pheasant in shared mid-mountain forest habitats, necessitating targeted conservation management in these critical overlap areas.

### 3.5. Change of Elliot’s Pheasant Habitat Under Climate Change

**Figure 5:**
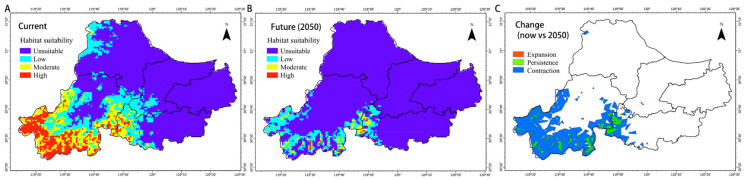
Distribution of suitable habitats for *Syrmaticus ellioti* under (**A**) current climate conditions and (**B**) 2050 climate conditions, and (**C**) change of suitable habitats between current and 2050 climate conditions.

Under current conditions, the suitable habitat for Elliot’s Pheasant (moderate and high suitability) is limited to approximately 1085.7 km^2^. By 2050, projections indicate severe habitat degradation and range contraction (Figure 5C). Suitable habitat area is predicted to plummet to 118.8 km^2^, representing a total loss of 84.6%. Alongside this reduction, the distribution center is expected to shift significantly. Future core areas are predicted to migrate from the current Anji Salamander National Nature Reserve toward southwestern Deqing and southeastern Anji. This drastic reduction and spatial shift will force Elliot’s Pheasant to adapt to shifting thermal gradients while simultaneously facing competition from sympatric species. Climate-driven changes in vegetation succession and the frequency of extreme temperatures will likely weaken habitat connectivity between isolated populations, threatening genetic diversity and long-term population stability.

## 4. Discussion

By integrating infrared camera monitoring data with MaxEnt modeling, this study systematically revealed the spatial distribution patterns of ground-dwelling pheasants in Huzhou and their response mechanisms to environmental factors. The results not only confirmed the dominant role of climatic stability and topographic factors in habitat selection but also predicted severe survival challenges for sensitive species, such as Elliot’s Pheasant, under future climatic fluctuations. These findings provide a scientific basis for developing targeted regional biodiversity conservation strategies.

### 4.1. Key Environmental Factors Influencing Distribution and Their Ecological Significance

Model simulations indicated that the potential distribution of Elliot’s Pheasant was driven by the synergistic effects of thermal stability, elevation, and seasonal moisture conditions. Iso-thermality (Bio03) emerged as the primary contributor (26.6%), consistent with research on species such as the Cheer Pheasant (*Catreus wallichii*), which emphasized the restrictive role of mean diurnal temperature range on habitat selection [18]. This reflects the high dependence of Elliot’s Pheasant on the stability of forest micro-climates.

Elevation (Alt) served as the second-largest contributor (18.0%) with high permutation importance (17.9%), defining a vertical distribution center between 200 and 600 m in the Huzhou region. This aligns with habitat selection patterns observed in the Brown Eared Pheasant (*Crossoptilon mantchuricum*) and Western Tragopan (*Tragopan melanocephalus*), which exhibit preferences for specific altitudinal gradients [19,20]. Elevation regulates the redistribution of heat and moisture, providing diverse vertical vegetation structures and concealment spaces. Furthermore, temperature annual range (Bio07, 13.2%) and mean temperature of driest quarter (Bio09, 6.4%) collectively influenced the distribution, underscoring the constraints imposed by extreme temperatures and seasonal thermal patterns. Notably, the 31.7% permutation importance of Bio09 revealed that the winter thermal baseline is critical for the stable presence of the species. Since our VIF analysis (VIF < 2) confirms that this result was not an artifact of multicollinearity, the high permutation importance highlights Bio09 as a genuine, critical limiting factor for the species’ physiological homeostasis. This shares a similar biological logic with the thermal habitat contraction observed in Blood Pheasants (*Ithaginis cruentus*) during wintering periods [21].

Regarding moisture factors, precipitation seasonality (Bio15, 14.7%) and precipitation of coldest quarter (Bio19, 5.5%) contributed significantly to the model. The seasonal distribution of precipitation indirectly determines food resource abundance during breeding and wintering stages by affecting the vitality of understory vegetation, such as ferns and shrubs. Although the contribution of the human footprint index (HFP, 4.3%) was slightly below the 5% threshold, the highly localized monitoring results reflected the species’ stringent requirements for primary habitat integrity. Coupled with findings that land-use change leads to habitat fragmentation for Reeves’s Pheasant (*Syrmaticus reevesii*) [22], the sensitive response of Elliot’s Pheasant to this combination of environmental factors highlights its pronounced vulnerability to future climate fluctuations and land-use shifts.

Furthermore, the impact of anthropogenic disturbance on habitat quality exhibited complexity across different scales. While some studies in North America suggest that landscape fragmentation has limited impact on the Common Pheasant (*Phasianus colchicus*) populations [23], in highly developed regions like Iran and China, road construction and the human footprint significantly exacerbate habitat loss by altering land-use structures [24]. Research on Reeves’s Pheasant warns that combined land-use and climate changes lead to a substantial decline in ecological corridor connectivity [22]. The high level of avoidance of anthropogenic areas observed in this study for Elliot’s Pheasant is consistent with habitat degradation patterns caused by human pathway interference in Mrs. Hume’s Pheasant (*Syrmaticus humiae*) [25] and the Brown Eared Pheasant [26].

### 4.2. Competitive Landscape and Collaborative Conservation Recommendations

Within the ground-dwelling pheasant community in Huzhou, spatial relationships and habitat selectivity differences are key to understanding stable coexistence or potential competition. Quantitative results showed that Elliot’s Pheasant occupied a passive position characterized by high competitive pressure and low resource occupancy. Ecological niche overlap analysis (Schoener’s D) indicated moderate-to-high niche sharing between Elliot’s Pheasant and its sympatric counterparts (D = 0.407 − 0.684). While niche equivalency tests suggested no significant differentiation among species pairs (*p* > 0.05), the similarity test revealed that the niche overlap with the Silver Pheasant significantly exceeded random expectations (*p* < 0.01), pointing toward niche convergence. These findings imply that Elliot’s Pheasant shares a highly similar realized niche with local pheasant species, with potential inter-specific competition most intense between Elliot’s Pheasant and the Silver Pheasant. This high spatial overlap suggests intense inter-specific competition, similar to the 90.78% spatial overlap observed between Blue Eared Pheasants (*Crossoptilon auritum*) and Blood Pheasants in the Qilian Mountains [21].

Within the ground-dwelling pheasant community in Huzhou, spatial relationships and habitat selectivity differences are key to understanding stable coexistence or potential competition. Quantitative results showed that Elliot’s Pheasant occupied a passive position characterized by high competitive pressure and low resource occupancy. This high degree of overlap, coupled with significant niche convergence with the Silver Pheasant, suggests intense inter-specific competition, similar to the 90.78% spatial overlap observed between Blue Eared Pheasants (*Crossoptilon auritum*) and Blood Pheasants in the Qilian Mountains [21].

However, the coexistence of sympatric pheasants often relies on fine-scale differentiation across multi-dimensional niches. For instance, the pheasant community in the Fanjingshan Reserve achieves occupancy avoidance through altitudinal gradients and vegetation preferences [14]. Research in the Qilian Mountains further revealed that species may alleviate competition through differing responses to NDVI thresholds or divergent monthly activity rhythms [21]. In contrast, Elliot’s Pheasant in this study—due to its strict iso-thermality requirements and preference for evergreen–deciduous broad-leaved mixed forests—was forced into core forest areas with minimal human interference, forming a typical “forced distribution”. Silver Pheasants and Chinese Bamboo Partridges, possessing broader ecological thresholds, occupied peripheral areas, severely compressing the living space of Elliot’s Pheasant.

Under the 2050 climate scenario, this competitive equilibrium faces a risk of collapse. The 84.6% habitat loss rate for Elliot’s Pheasant far exceeds that of large mammals in the same region, predicting a severe population bottleneck. As thermal gradients shift, the core distribution area is expected to move toward Mogan Mountain in Deqing and southeastern Anji. This process involves not only horizontal spatial displacement but also the need for the species to relocate suitable altitudinal bands. To address these dual pressures, future conservation strategies should be more targeted and systemic. Firstly, precise maintenance of core micro-habitats and spatial heterogeneity. Priority should be given to reinforcing the structural integrity of evergreen–deciduous broad-leaved mixed forests within the 200–600 m altitudinal band. Given the species’ reliance on microclimate stability, increasing under-story canopy cover and strictly controlling forest-edge interference can preserve critical refugia for Elliot’s Pheasant during resource competition with dominant species. Secondly, proactive layout of ecological corridors for future suitable areas. Considering the eastward shift of the distribution center, restoration of landscape connectivity from southeastern Anji to western Deqing should be initiated. Following recommendations to strengthen forest connectivity for Blue Eared and Blood Pheasants [21], establishing protected migration pathways will assist Elliot’s Pheasant in cross-regional shifts driven by climate change, mitigating the risk of isolation caused by habitat fragmentation. Furthermore, establishment of multi-species synergistic and dynamic monitoring. Conservation efforts should focus on a systemic assessment of the entire mid-mountain forest pheasant community. Continuous dynamic monitoring in high-overlap areas, combined with occupancy modeling, can identify risk points where competitive pressure surges. By dynamically adjusting protection boundaries and habitat interventions, the stable reproduction of Elliot’s Pheasant can be ensured, preventing it from being entirely marginalized during community succession.

### 4.3. Study Limitations

Several limitations should be considered when interpreting our findings. First, our climate change projections were based exclusively on the SSP2-4.5 intermediate scenario. While this represents a plausible “middle-of-the-road” pathway, it does not fully encompass the range of potential climatic uncertainties, such as the more optimistic SSP1-2.6 or the more severe SSP5-8.5 scenarios. Consequently, our projections should be viewed as a conservative baseline rather than an exhaustive range of potential impacts.

Second, although we included essential bioclimatic and topographic variables, we prioritized a parsimonious model approach to avoid severe multicollinearity and overfitting. While more complex topographic indices (e.g., topographic position index and topographic wetness index) could further describe fine-scale microhabitats, we limited our selection to primary environmental predictors to ensure model stability and biological interpretability. Future studies incorporating a broader spectrum of climate scenarios and finer-scale microhabitat variables would provide a more comprehensive understanding of the species’ sensitivity to environmental changes, ultimately facilitating more precise conservation management.

## 5. Conclusions

This study confirmed that Elliot’s Pheasant exists in an extremely sensitive and high-pressure competitive state within the Huzhou pheasant community. Its core habitat is highly dependent on evergreen broad-leaved mixed forests at 200–600 m elevation, characterized by stable iso-thermality and minimal disturbance. Niche analysis showed that its habitat is nearly entirely subsumed by Silver Pheasants and Chinese Bamboo Partridges, with a high spatial overlap with Koklass Pheasants, indicating intense spatial compression. Projections for 2050 suggest an 84.6% habitat loss and a significant eastward shift. Therefore, future conservation focus must transition from traditional in-situ protection to cross-regional corridor construction and multi-species synergistic monitoring to address population bottleneck risks posed by climate change.

## Figures and Tables

**Table 1:** Important ecological factors adopted for terrestrial pheasants in the models. The AUC is the mean value ± SD across 10 replicates.

*Syrmaticus ellioti* AUC = 0.963 ± 0.003			*Lophura nycthemera* AUC = 0.909 ± 0.002			*Pucrasia macrolopha* AUC = 0.961 ± 0.001			*Bambusicola thoracicus* AUC = 0.859 ± 0.007		
Variables	Percentage Contribution	Permutation Importance	Variables	Percentage Contribution	Permutation Importance	Variables	Percentage Contribution	Permutation Importance	Variables	Permutation Importance	Permutation Importance
Bio03	26.6	4.8	Bio03	33	29.1	Bio03	21.8	12.5	Bio07	27.1	5.9
Alt	18	17.9	Bio07	19.7	9.9	Bio09	20.2	18.7	Bio09	22.5	19.2
Bio15	14.7	7.8	Bio09	15.4	14.6	Bio06	12.1	2.9	Bio17	12.6	10
Bio07	13.2	3.6	Bio15	6.4	3.8	Bio15	11.4	11	Bio03	8.6	7.2
Bio09	6.4	31.7	Alt	6.2	7.5	Bio07	11.1	22.4	Bio12	5.9	11.3
Bio19	5.5	11.1	Bio19	6	16.2	Alt	9.6	4.1	Alt	1.7	19.8
HFP	4.3	0.8	Bio12	1.1	4.9	Bio17	3.6	5.6			

**Table 2:** Comparison of current suitable habitats and core ecological factor suitability thresholds for four pheasants in Huzhou.

	Habitat	*Syrmaticus ellioti*	*Lophura nycthemera*	*Pucrasia macrolopha*	*Bambusicola thoracicus*
Habitat area (km^2^)	Unsuitable	3820.0	2957.7	3932.7	1359.6
	Low suitability	913.1	796.4	575.6	783.7
	Moderate suitability	652.1	901.3	446.0	1398.4
	High suitability	434.8	1164.6	865.7	2278.3
Ecological factor	Alt	200–600 m	<500	600–1000 m	<500
	Bio9	2 °C	--	1 °C	3.5 °C
	Bio3	22–26 °C	--	--	--
Spatial expansion capability		Weak, most specialized habitat requirements	Strong, dominant in the mid-mountain belt	Moderate, strictly confined to high-altitude vertical zones	Strong, widely penetrating into low mountains and hills

Note: Some parameters were left blank because the contribution of this factor in the corresponding species’ model was low (not in the top five or the response curve was not significant) and thus it is not listed as a core threshold.

## Data Availability

Dataset available on request from the authors.

## Supplementary Materials

The following Supporting Information can be downloaded at https://www.mdpi.com/article/10.3390/biology15060480/s1, Figure S1. Maxent model analysis results and the influence of related ecological factors for three other pheasants.
