# Supplementary figures and images for: Impacts of Climate Change and Inter-Specific Competition on the Spatial Distribution of Elliot’s Pheasant (Syrmaticus ellioti, Swinhoe, 1872) in Huzhou City, China
Source: Biology (Basel). 2026 Mar 18;15(6):480. doi: 10.3390/biology15060480 (PMC13024169; doi:10.3390/biology15060480)

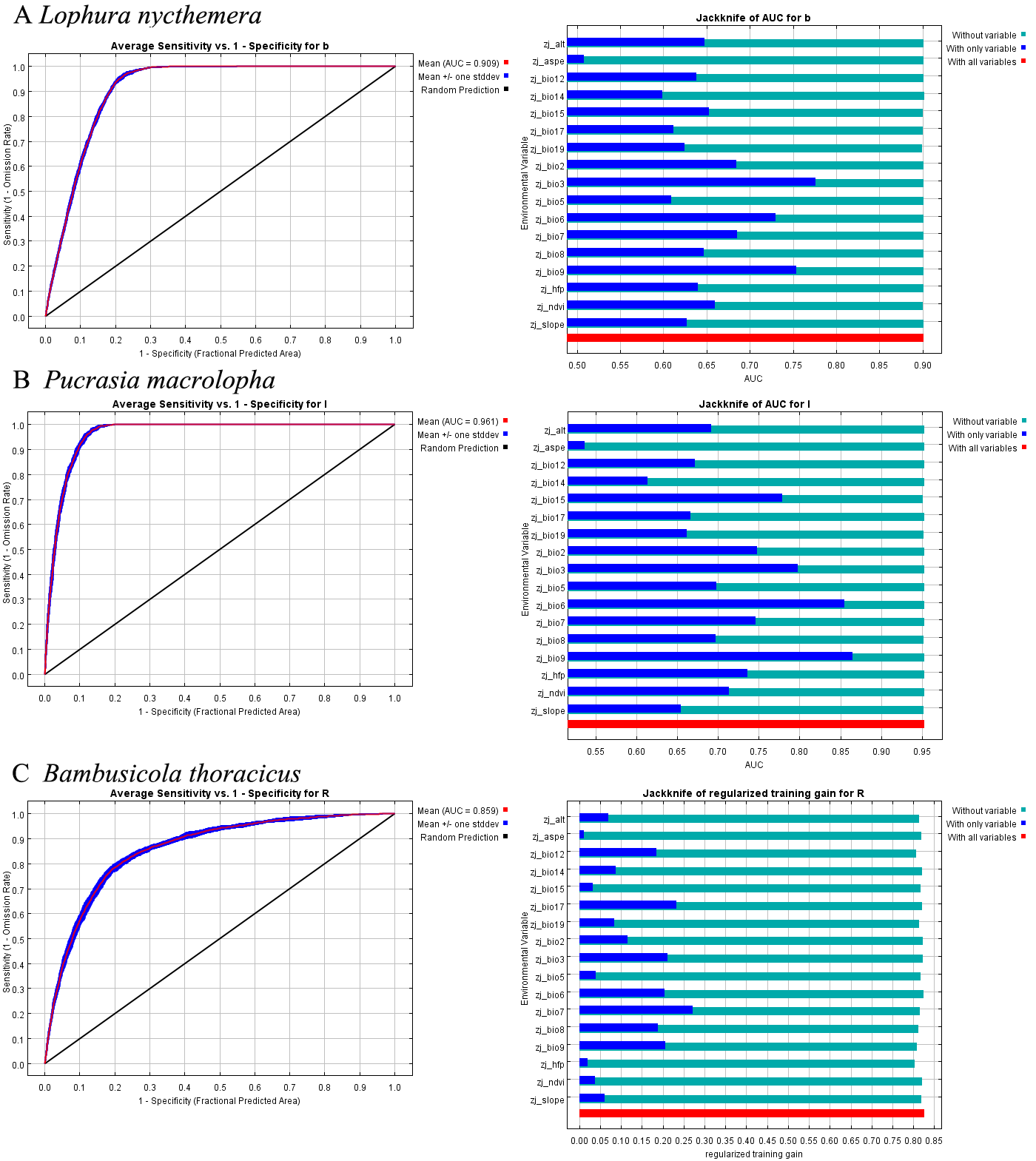

Supplement: Supplementary file 1 [file biology-15-00480-s001.zip › biology-4208952-Figure S1.tif]
